# Proteomic Profiling of *Medicago sativa* Leaves upon *Spodoptera litura* (Lepidoptera: Noctuidae) Invasion: Activation of Defense Pathways and Photosynthetic Resource Reallocation

**DOI:** 10.3390/plants15193064

**Published:** 2026-10-07

**Authors:** Lei Xu, Yujie Li, Ruixin Zhang, Richard John Tiika, Mingna Li, Yong Wang, Fang Wu, Huirong Duan, Ruicai Long, Hongshan Yang

**Affiliations:** 1 Lanzhou Institute of Husbandry and Pharmaceutical Sciences, Chinese Academy of Agricultural Sciences, Lanzhou 730050, China; xulei05@caas.cn (L.X.); lyj230712@126.com (Y.L.); 18894019164@163.com (R.Z.); tiikarichard@gmail.com (R.J.T.); wangyong05@caas.cn (Y.W.); wufang@caas.cn (F.W.); duanhuirong@caas.cn (H.D.); 2 Lanzhou Loess Plateau Ecological Environment Key Field Scientific Observation and Experiment Station, Ministry of Agriculture and Rural Affairs, Lanzhou 730050, China; 3 Institute of Animal Sciences, Chinese Academy of Agricultural Sciences, Beijing 100193, China; limingna@caas.cn

**Keywords:** *Medicago sativa*, proteomic analysis, *Spodoptera litura*, phenylpropanoid, flavonoid, photosynthesis

## Abstract

Alfalfa (*Medicago sativa* L.) is a high-quality perennial leguminous forage that plays vital roles in livestock industry development and ecological restoration. However, *Spodoptera litura* (Fabricius, 1775) (Lepidoptera: Noctuidae) severely impairs alfalfa yield and quality. At present, systematic proteomic analyses characterizing the expression profiles and regulatory networks of key insect-resistant proteins under herbivory stress remain limited. The objective of this study is to identify the critical anti-insect factors and resolve the regulatory networks controlling alfalfa adaptation to herbivory. In this study, leaf proteomes were compared between plants infested by *S. litura* for 24 h and non-infested controls. A total of 6427 proteins were identified, including 586 differentially abundant proteins (DAPs). Functional enrichment analyses revealed that these DAPs were significantly enriched in pathways such as phenylpropanoid biosynthesis, flavonoid biosynthesis, α-linolenic acid metabolism and photosynthesis. Herbivory by *S. litura* increased the abundance of proteins associated with the phenylpropanoid pathway (e.g., PAL, 4CL, and COMT) and core enzymes involved in jasmonic acid (JA) biosynthesis (e.g., LOX2S, AOC, and OPR), whereas multiple photosynthesis-related proteins (e.g., psaB, petE, and ATPF0B) were decreased in abundance. This study elucidates the regulatory network governing insect defense in alfalfa at the proteomic level, providing valuable protein targets and theoretical support for molecular breeding of insect-resistant alfalfa.

## 1. Introduction

The leguminous forage crop alfalfa (*Medicago sativa* L.) has a wide cultivation distribution and outstanding nutritional value for livestock feed, thus occupying an important strategic position in the global livestock feed supply system. This species yields high biomass per unit area with a stable dry-hay crude protein content range of 15–25% while providing multiple ecological services including biological nitrogen fixation, soil improvement, and water–soil conservation [1]. In recent years, China’s alfalfa self-supply capacity has improved significantly, but it still lags behind in excellent germplasm resources, self-breeding level, and large-scale promotion. Currently, the agronomic management of alfalfa cultivation is relatively complete, but pests still occur widely in various planting areas, continuously causing yield losses and quality deterioration. Common alfalfa pests include alfalfa cutworms (Lepidoptera: Noctuidae), aphids (Hemiptera: Aphididae), thrips (Thysanoptera: Thripidae), alfalfa weevil (*Hypera postica*), potato leafhopper (*Empoasca fabae*), etc., and their harm spans the entire growth cycle of alfalfa [2]. *S. litura* is a broad-spread and voracious pest with migratory ability. Its larvae can harm over 100 species of plants and is widely distributed in tropical and subtropical regions of Asia. In China, it is mainly found along the Yangtze and Yellow River basins and southern provinces [3]. The larvae of this pest cause leaf damage via chewing feeding, and heavy outbreaks can lead to complete host defoliation, seriously impairing the yield and quality of economically important crops such as peanuts and alfalfa [4,5]. The extensive application of chemical pesticides in alfalfa production has led to pesticide resistance in herbivorous insect pests, together with ecological risks and environmental pollution. So breeding for natural genetic resistance is key to achieving both productivity and ecological safety.

In response to the pressure from plant-eating insects, plants have built a defense system over time. This system has many layers. It has physical barriers, chemical weapons, and signals that move between local and distant tissues [6]. For example, trichomes, cuticles, and lignified tissues make leaves harder for pests to attach to and eat. Biochemically, secondary metabolites (e.g., terpenoids, phenolics, and alkaloids) together with protease inhibitors disrupt insect digestion or exert toxicity. Ecologically, herbivore-induced volatiles recruit natural enemies as an indirect defense. Crucially, these layers are not merely additive but are synergistically coordinated via phytohormonal crosstalk [7]. Among these traits, the absence of glandular trichomes and waxes markedly increases thrips susceptibility in alfalfa varieties [8]. Wild tomato (*Solanum habrochaites*) glandular trichomes defend against insects through physical barriers, chemical defenses, and Ca^2+^-triggered jasmonate signaling that drives local and systemic resistance upon herbivore touch [9]. Plant chemical defense against herbivory relies on a synergistic repertoire of anti-herbivore factors, including secondary metabolites and defensive proteins (e.g., protease inhibitors and lectins), among which overexpression of the trypsin inhibitor *TaBBTI* enhances broad-spectrum resistance to pests [10,11]. Insecticidal proteins deployed through transgenic approaches are central to engineered plant protection, with *Bacillus thuringiensis*-derived Vip3Aa being particularly noteworthy for its broad deployment in transgenic maize and cotton against lepidopteran pests [12]. iJAZ proteins containing the NGR motif confer transgenic resistance to multiple lepidopteran pests, including *Spodoptera frugiperda*, *Helicoverpa armigera*, and *Cnaphalocrocis medinalis*, thereby expanding the repertoire of insect-resistance genes for crop improvement [13]. Meanwhile, the adoption of fusion protein approaches is on the rise. As a typical example, the Cry1Ac-ASAL fusion structure has been proven to be able to effectively protect rice from damage caused by lepidopteran and hemipteran pests, thereby increasing yields [14].

In terms of signal regulation, the JA signaling pathway constitutes the main defense mechanism of plants against chewing pests. Among them, the JAZ inhibitory factor, the F-box protein COI1, and the MYC transcription factor are key molecular nodes that regulate signal transduction and transcriptional activation [15]. After plants perceive herbivorous pests, they induce jasmonoyl-L-isoleucine (JA-Ile) accumulation and release MYC2 transcription factors via COI1-dependent JAZ degradation, thereby activating downstream defense genes [6]. Lipoxygenase (LOX) catalyzes a key step in the biosynthesis of JA and plays a crucial role in regulating plant defense responses. Its expression level and enzyme activity are directly related to the adaptive response of plants to biological stress, including insect feeding and pathogen infestation [16]. Knocking out the core regulatory factor COI1 in the JA signaling pathway significantly reduces the expression of TrypPI, POD, and PPO [17]. In addition, the interaction between the JA pathway and other hormone signaling pathways such as SA, ET, and BR forms a complex regulatory network that can precisely regulate plant defense responses [18]. However, the proteomic landscape of JA-triggered defense in *S. litura*-infested alfalfa remains poorly studied; downstream mobilization proteins, tissue-specific modifications, and regulatory networks are still unclear. The phenylpropanoid pathway acts as a complementary defense network, producing lignin for structural reinforcement and flavonoids for biochemical inhibition, which together form a multidimensional defense against chewing pests [19,20]. The rapid invasion of herbivorous insects can quickly induce the expression of PAL, C4H, and 4CL, directing carbon flow towards the synthesis of lignin and flavonoids. Lignin deposition regulated by COMT, CCoAOMT, CCR, and CAD synergistically enhances the cell wall for physical defense; the generation of flavonoids driven by F3H and FLS interferes with insect digestion processes or produces toxic effects [9,21]. The cassava *MeCHS* gene exhibits coordinated transcriptional and proteomic responses to the infestation of the spider mite *Tetranychus cinnabarinus*, highlighting its key role in spider-mite resistance [22]. Most previous studies have provided valuable insights at the gene transcription level, but the protein-level regulation of these key enzymes under pest invasion has received relatively less attention.

Integrating multi-omics provides a powerful framework for elucidating plant insect defense networks. Joint analysis of alfalfa showed that adult alfalfa weevils preferentially induce the phenylpropanoid or benzenoid pathway during feeding, while larvae mainly activate the JA pathway during feeding [23]. Integrated transcriptomic and metabolomic analysis of wild and cultivated wheat reveals strong genotypic divergence, especially in genes for primary metabolic enzymes, hormone signaling mediators, and defense-related specialized metabolites [24]. Combined transcriptomic and metabolomic analyses show that flavonoid and isoflavonoid biosynthesis was the most responsive pathway to thrips infestation and that SA and JA signaling, PTI, and MAPK cascades cooperatively regulated the induced resistance in alfalfa [25]. Proteomic analyses of rice, *Arabidopsis*, and tomato responses to their respective insect pests, namely *Laodelphax striatellus*, *Plutella xylostella*, and *Macrosiphum euphorbiae*, have consistently implicated differentially abundant proteins in stress defense, photosynthesis, cell wall modification, and ROS metabolism. These studies further highlight ROS imbalance and the synergistic activation of the SA, JA, ET, and auxin pathways as shared signatures of effective resistance [26]. Proteomic analyses of the chickpea–cotton bollworm system show that resistant cultivars defend via JA signaling, fatty acid metabolic flux, and transcriptomic remodeling, while the pest counteracts with elevated detoxification and proteolytic activity [27]. Nevertheless, given that proteins are the proximate effectors of defense, their abundance, PTMs, and interactomic dynamics are often inaccurately predicted from transcriptional profiles. Moreover, modification-based proteomics in rice has shown that insect feeding upregulates lysine malonylation, predominantly in high-abundance proteins, which cooperatively fine-tunes the transcription of host defense-related genes [28].

Transcriptomic and metabolomic profiling in our previous work has established that *S. litura* infestation activates both the phenylpropanoid and JA biosynthetic cascades in alfalfa, culminating in the deployment of anti-herbivore defenses [29]. Nevertheless, mRNA dynamics often diverge from protein-level changes, leaving the functional protein networks governing signal transduction, metabolic flux, and photosynthetic adjustment largely unresolved. Here, we applied label-free quantitative proteomics to profile the leaf proteome of alfalfa in response to *S. litura* infestation. Our specific objectives were to verify and extend our prior transcriptomic findings at the protein translational level and to uncover herbivory-responsive protein regulatory networks that are undetectable by transcriptome analysis alone. Beyond validating previously reported defense pathways, this proteomic resource further enables the discovery of candidate functional proteins, which can serve as promising molecular targets for future insect-resistance breeding in alfalfa.

## 2. Results

### 2.1. Data Quality Analysis of Proteomic Analysis

**Figure 1:**
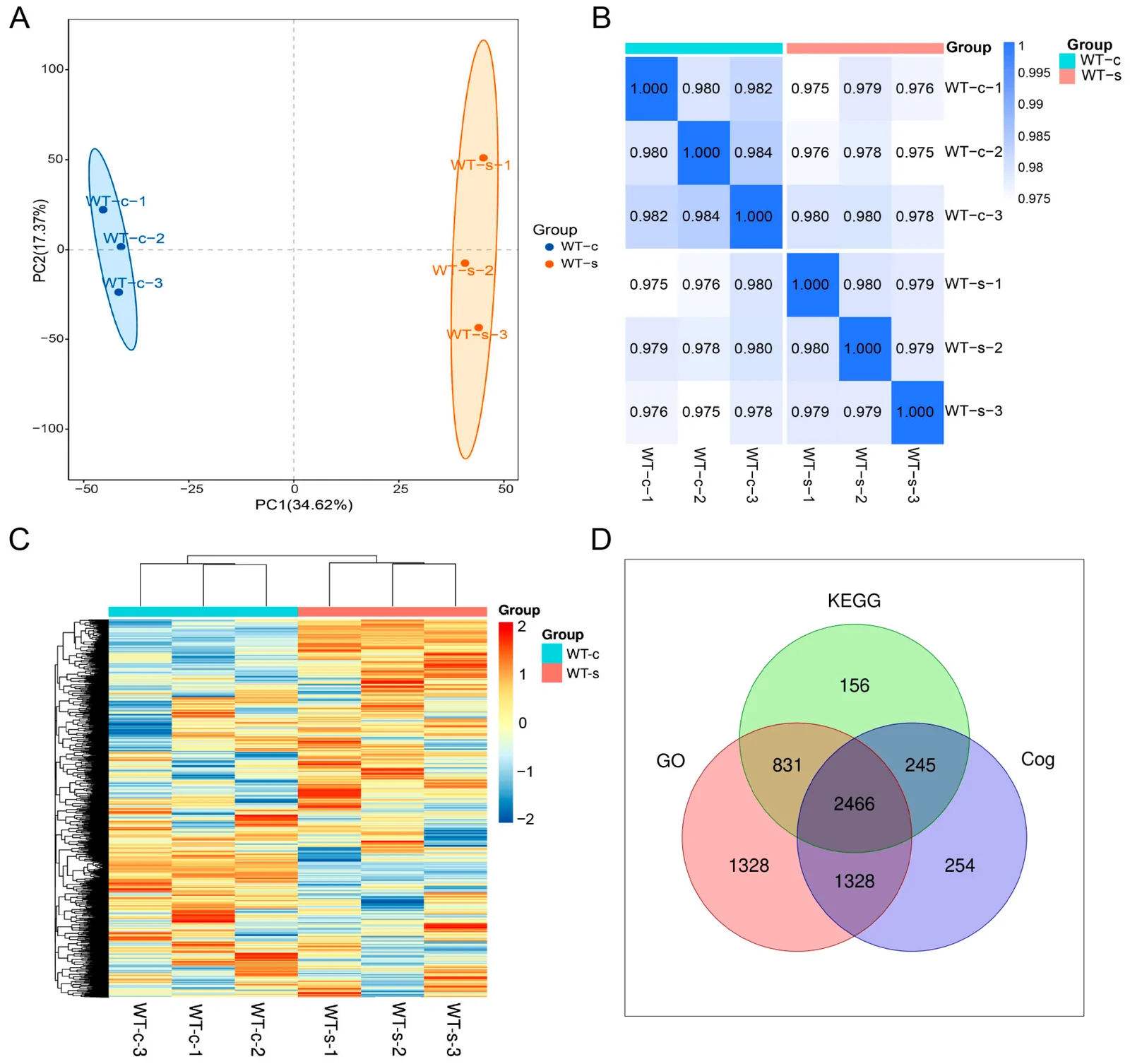
Protein sample global profiling and functional annotation analysis. (**A**) Principal Component Analysis (PCA) of all protein samples. The *x*-axis and *y*-axis represent the first and second principal components, respectively; the percentage values labeled on each axis stand for the contribution rate of the corresponding principal component to the total variance of all samples. Each dot represents a single sample. Samples from identical experimental groups are assigned the same color, whereas different groups are differentiated by disparate colors. (**B**) Correlation heatmap of expression levels across all protein samples. Pearson’s Correlation Coefficient (r) closer to 1 indicates stronger correlation between any two samples. (**C**) Expression heatmap of all protein samples. Each column represents a sample, and each row represents a protein. Different colors denote varying expression levels, with orange indicating high expression and blue indicating low expression. (**D**) Venn diagram for the statistical results of protein functional annotation, displaying the counts of proteins uniquely annotated in the GO, KEGG, COG databases and the overlapped annotated proteins among these databases.

To investigate the roles of key proteins of alfalfa in its defense against chewing insects, a feeding experiment was conducted using *S. litura* in alfalfa. Proteomic analysis was performed on alfalfa leaf samples under two conditions, namely without *S. litura* feeding (WT-c: WT-c-1, WT-c-2, and WT-c-3) and following 24 h of *S. litura* feeding (WT-s: WT-s-1, WT-s-2, and WT-s-3), with three biological replicates per group. A total of 6427 proteins were identified (Supplementary Table S1). Principal component analysis (PCA) clearly segregated all protein samples into two major clusters, corresponding to non-infested controls and *S. litura*-infested alfalfa samples, which reflected distinct global protein abundance profiles between the two groups (Figure 1A). The correlations between the two groups of protein samples are visualized using a correlation heatmap (Figure 1B), and the expression profiles of all identified proteins are depicted in a global heatmap (Figure 1C). Among the identified proteins, 5953 (92.6%), 3698 (57.5%), and 4293 (66.8%) were annotated in the Gene Ontology (GO), Kyoto Encyclopedia of Genes and Genomes (KEGG), and Clusters of Orthologous Groups (COG) databases, respectively, with 2466 (34.6%) proteins co-annotated by all three databases (Figure 1D).

### 2.2. Analysis of Expression Patterns and Subcellular Localization of Differentially Abundant Proteins

**Figure 2:**
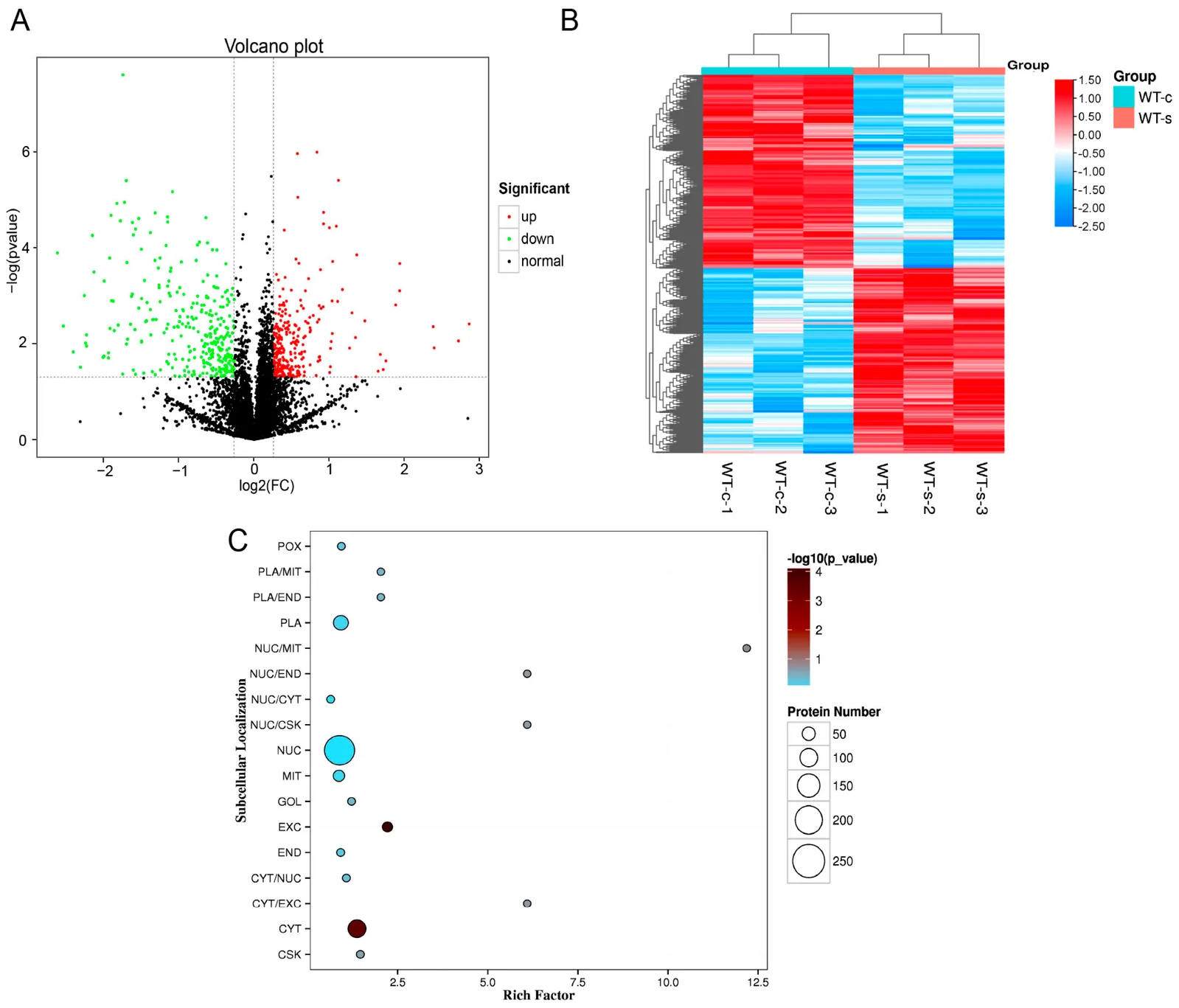
Identification of DAPs. (**A**) Volcano plot displaying the distribution of DAPs. The *x*-axis represents the log2 fold change (FC) of the quantitative ratio between group samples, and the *y*-axis represents the -log2 of the *t*-test *p*-value. Red indicates upregulated proteins, while green indicates downregulated proteins. (**B**) Hierarchical clustering heatmap of DAP expression profiles. Columns represent individual samples, and rows correspond to distinct DAPs. Clustering is based on log_2_-transformed protein expression values, with red representing highly expressed proteins and green representing lowly expressed proteins. (**C**) Bubble plot of subcellular localization enrichment for DAPs. The *x*-axis represents the rich factor, and the *y*-axis indicates subcellular localization. The size of each bubble corresponds to the number of enriched DAPs, and a deep red color corresponds to greater enrichment significance. CSK: cytoskeleton; CYT: cytoplasm; END: endoplasmic reticulum; EXC: extracellular; GOL: Golgi apparatus; MIT: mitochondrion; NUC: nucleus; PLA: plasma membrane; POX: peroxisome.

In this study, a total of 586 DAPs were identified from six samples. Following feeding by *S. litura*, 286 DAPs were upregulated and 300 were downregulated compared with the non-infested control plants (Figure 2A and Supplementary Table S2). Meanwhile, heatmap clustering of the specific expression patterns of these DAPs illustrated protein-abundance profiles under *S. litura* feeding, as well as sample clustering and good consistency among biological replicates (Figure 2B).

To dissect the distribution characteristics of defense proteins in alfalfa in response to feeding by *S. litura* at the subcellular level, subcellular localization prediction was performed on all DAPs in this study. The results revealed that DAPs were widely distributed across multiple subcellular compartments. The top four compartments with the largest number of localized proteins were the nucleus (NUC, 264, accounting for 45.05% of total DAPs), cytoplasm (CYT, 122, accounting for 20.82%), plasma membrane (PLA, 85, accounting for 14.51%), and mitochondrion (MIT, 46, accounting for 7.85%), respectively. This indicates that the defense response of alfalfa to insect herbivory involves multi-layered processes including transcriptional regulation (nucleus), signal transduction (cytoplasm), signal perception (plasma membrane), and energy reprogramming (mitochondria). Subcellular localization enrichment analysis demonstrated that DAPs were significantly enriched in several cellular compartments. The enrichment levels of CYT and the extracellular space (EXC) were the highest, indicating that under herbivorous stress, there were specific and significant differential expressions of cytoplasmic proteins and secreted proteins (Figure 2C). Overall, the discovery of subcellular localization and cellular component enrichment analysis provides key spatial localization clues for further dissecting functional protein pathways.

### 2.3. Enrichment Analysis of Differentially Abundant Proteins

**Figure 3:**
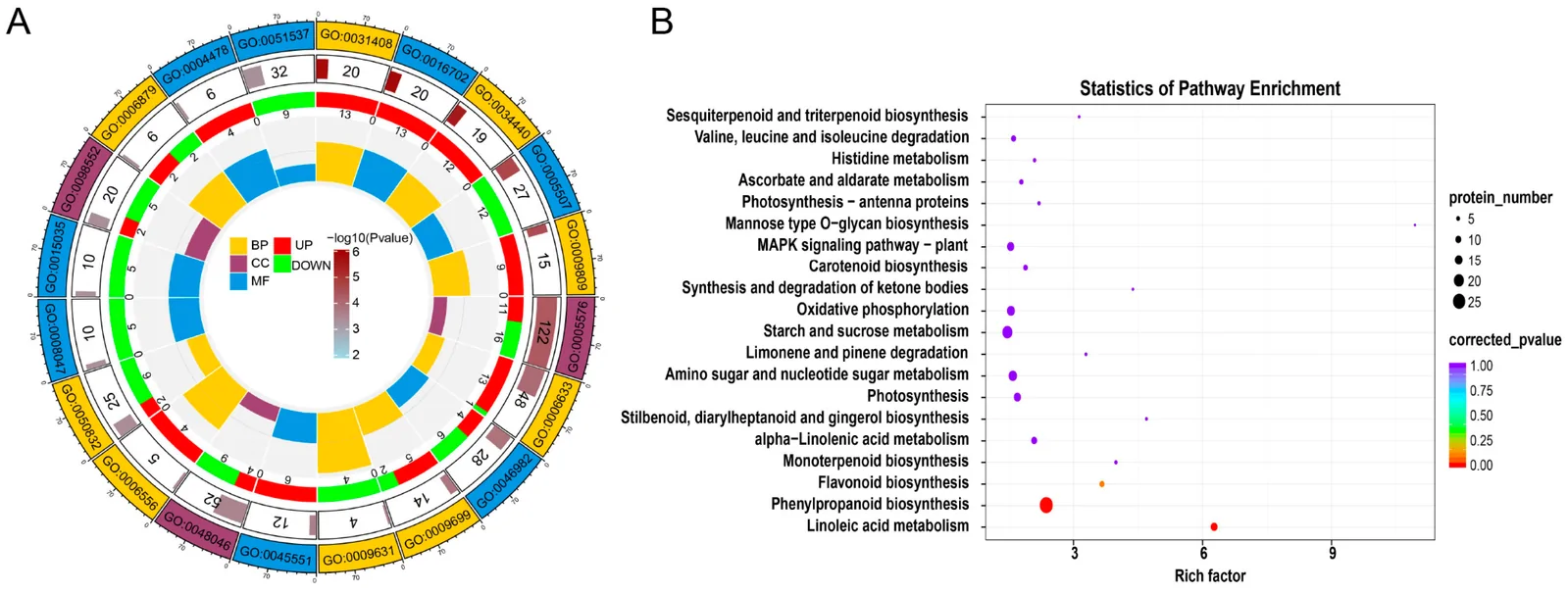
Functional enrichment profiling of DAPs. (**A**) GO enrichment circle plot of DAPs. The first ring indicates the GO term identifiers. The second ring represents the number of all DAPs annotated to each GO term, with color intensity reflecting the significance of DAP enrichment (darker shades denote higher significance). The third ring shows the number of DAPs enriched in each GO term, with red and green bars denoting upregulated and downregulated DAPs, respectively. The fourth ring indicates the proportion of DAPs relative to the total proteins annotated to the corresponding GO term. (**B**) KEGG pathway enrichment bubble plot of DAPs. Each bubble represents a KEGG pathway, with the vertical axis showing pathway names and the horizontal axis representing the enrichment factor. The size of bubbles represents the number of DAPs enriched in this pathway. The color of the bubbles represents the corrected *p*-value. The redder the color, the smaller the corrected *p*-value and the greater the significance of pathway enrichment.

Through GO annotation analysis of DAPs, a total of 36 GO categories were obtained. These GO categories primarily included the biological process, cellular component, and molecular function. Among these, the biological process category enriched the largest number of DAPs, followed by the molecular function category. Within the biological process category, the metabolic process and cellular process accounted for the highest proportions. In the molecular function category, binding and catalytic activity were the most prevalent. Within the cellular component category, the cellular anatomical entity and intracellular entity exhibited the highest proportions (Figure S1A). Among the top 20 significantly enriched GO categories of DAPs (including the lignin biosynthetic process (GO:0009809), the extracellular region (GO:0005576), the fatty acid biosynthetic process (GO:0006633), protein heterodimerization activity (GO:0046982), etc.), the biological process category enriched the greatest number of DAPs (Figure 3A; Supplementary Table S3).

We performed KEGG enrichment analysis on all DAPs, and the results showed that after feeding by *S. litura*, the DAPs in alfalfa were mainly enriched in 100 KEGG pathways (including both significantly and non-significantly enriched terms). The top 20 enriched pathways were mapped and analyzed (Figure 3B; Supplementary Table S4), which primarily included phenylpropanoid biosynthesis; flavonoid biosynthesis; monoterpenoid biosynthesis; alpha-linolenic acid metabolism; stilbenoid, diarylheptanoid and gingerol biosynthesis; photosynthesis; amino sugar and nucleotide sugar metabolism; starch and sucrose metabolism; oxidative phosphorylation; limonene and pinene degradation; carotenoid biosynthesis; and the MAPK signaling pathway. Among these, the KEGG pathways with downregulated DAPs mainly comprised photosynthesis, photosynthetic antenna proteins and limonene and pinene degradation (Figure S2A). The KEGG pathways with upregulated DAPs mainly included flavonoid biosynthesis, alpha-linolenic acid metabolism, and monoterpenoid biosynthesis (Figure S2B).

### 2.4. COG Database Annotation and Analysis of Differentially Abundant Proteins

Based on the COG database annotation, among the 586 identified DAPs, 396 DAPs (approximately 67.6%) were successfully assigned to COG functional categories. These proteins were classified into 26 functional groups, covering major biological processes such as information storage and processing, cellular metabolism, and signal transduction. Among them, the three most abundantly represented functional categories were posttranslational modification, protein turnover, chaperones [O] (55 DAPs, accounting for 13.9% of the annotated proteins), carbohydrate transport and metabolism [G] (49 DAPs, 12.4%), and general function prediction only [R] (39 DAPs, 9.8%). In addition, substantial numbers of proteins were also distributed in categories including translation, ribosomal structure and biogenesis [J] (32 DAPs, 8.1%) and secondary metabolite biosynthesis, transport and catabolism [Q] (30 DAPs, 7.6%) (Figure S1B). Collectively, the COG annotation results revealed that under this treatment condition, the major affected processes were protein homeostasis maintenance, central carbon metabolic pathways, translational machinery, and secondary metabolic networks.

### 2.5. Analysis of Differentially Abundant Proteins of Alfalfa Related to Phenylpropanoid and Flavonoid Biosynthesis During S. litura Feeding

**Figure 4:**
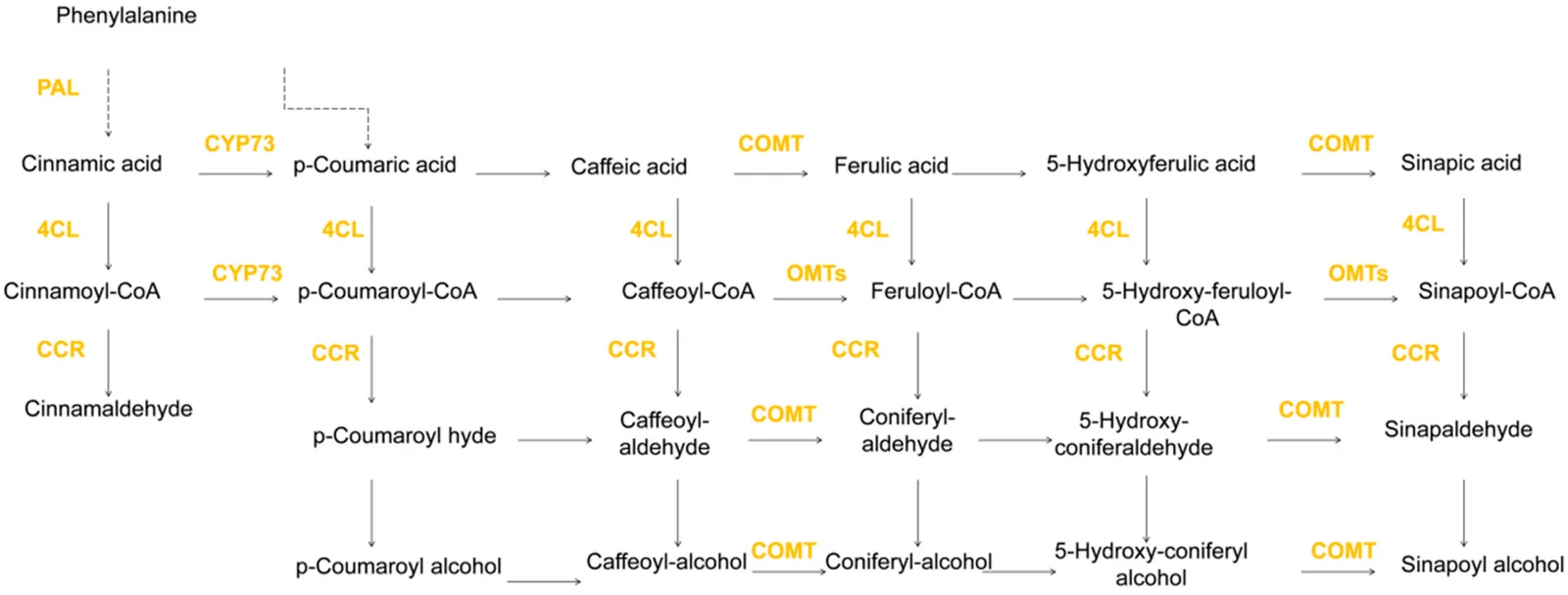
Schematic diagram of the phenylpropanoid biosynthesis pathway and DAPs in alfalfa. The yellow labeled proteins represent DAPs involved in this pathway. Solid arrows indicate single-step enzymatic reactions catalyzed by enzymes labeled in yellow. Dashed arrow represents multi–step reactions with intermediate metabolites omitted from the diagram. PAL: phenylalanine ammonia-lyase; CYP73A: trans–cinnamate 4–monooxygenase; 4CL: 4–coumarate:CoA ligase; COMT: caffeic acid 3–O–methyltransferase; OMTs: caffeoyl–CoA O–methyltransferase; CCR: cinnamoyl–CoA reductase.

**Figure 5:**
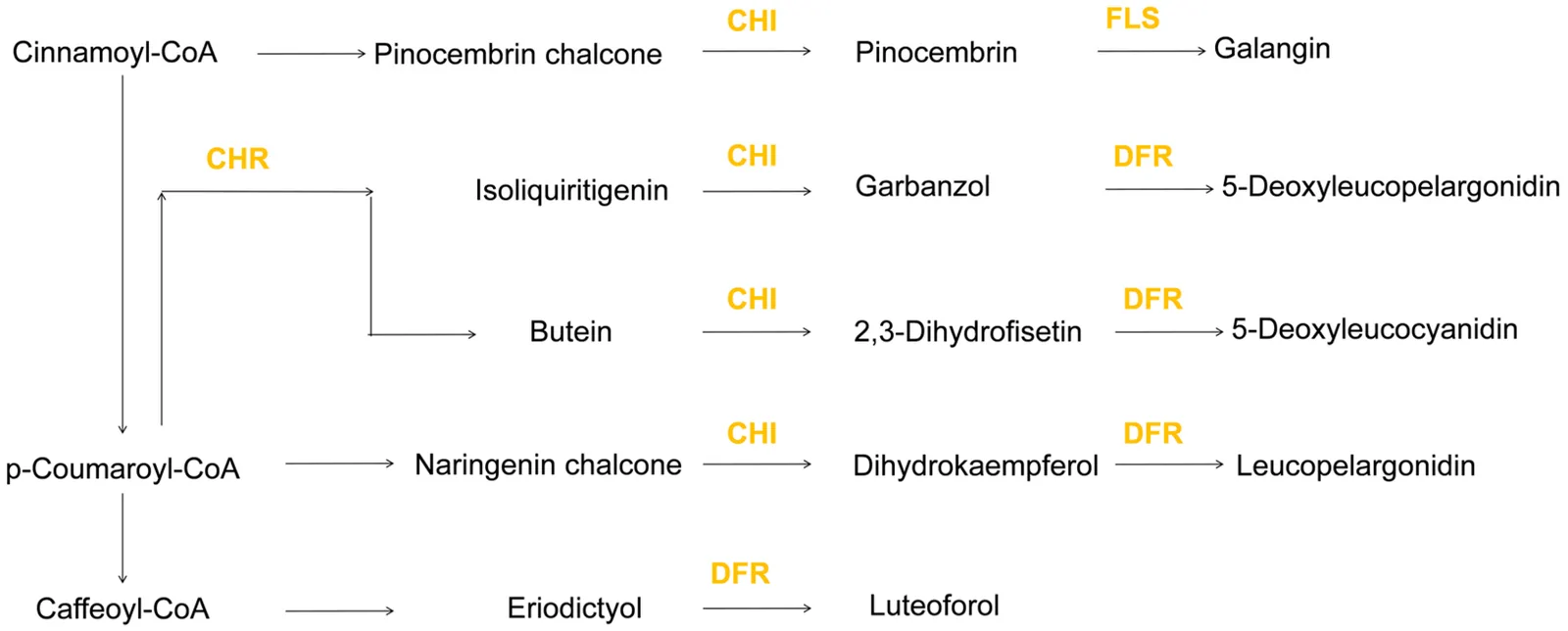
Schematic diagram of the flavonoid biosynthesis pathway and DAPs in alfalfa. The yellow labeled proteins represent DAPs involved in this pathway. Solid arrows indicate single–step enzymatic reactions catalyzed by enzymes labeled in yellow. CHR: chalcone reductase; CHI: chalcone isomerase; FLS: flavonol synthase; DFR: dihydroflavonol–4–reductase.

Through proteomic analysis and KEGG enrichment analysis, the DAPs between WT-c and WT-s were significantly enriched in phenylpropanoid-related pathways, including the phenylpropanoid biosynthesis (Figure 4) and flavonoid biosynthesis (Figure 5) pathways. The coordinated expression of enzyme proteins within these pathways mediates the synthesis of defensive compounds, such as lignin and flavonoids, via phenylpropanoid metabolic flux, implying potential alterations in corresponding metabolite pools upon *S. litura* infestation. In particular, the DAPs involved in phenylpropanoid biosynthesis included phenylalanine ammonia-lyase (PAL, A0A072U4G3), trans–cinnamate 4–monooxygenase (CYP73A, P37114), caffeic acid 3–O–methyltransferase (COMT, I3SR79), caffeoyl-CoA O-methyltransferase (OMTs, Q40313), and cinnamoyl-CoA reductase (CCR, A0A072VDF2). Those associated with flavonoid biosynthesis comprised chalcone reductase (CHR, Q40333), chalcone isomerase (CHI, A0A072VGT5), flavonol synthase (FLS, G7JBS9), and dihydroflavonol–4–reductase (DFR, G7IPP8). Compared with the control group, the levels of these proteins increased by 1.26- to 1.82-fold after *S. litura* feeding (Supplementary Table S5), indicating that herbivory by *S. litura* increased the abundance of proteins related to phenylpropanoid biosynthesis.

### 2.6. Analysis of Differentially Abundant Proteins of Alfalfa Related to Alpha-Linolenic Acid Metabolism During S. litura Feeding

**Figure 6:**
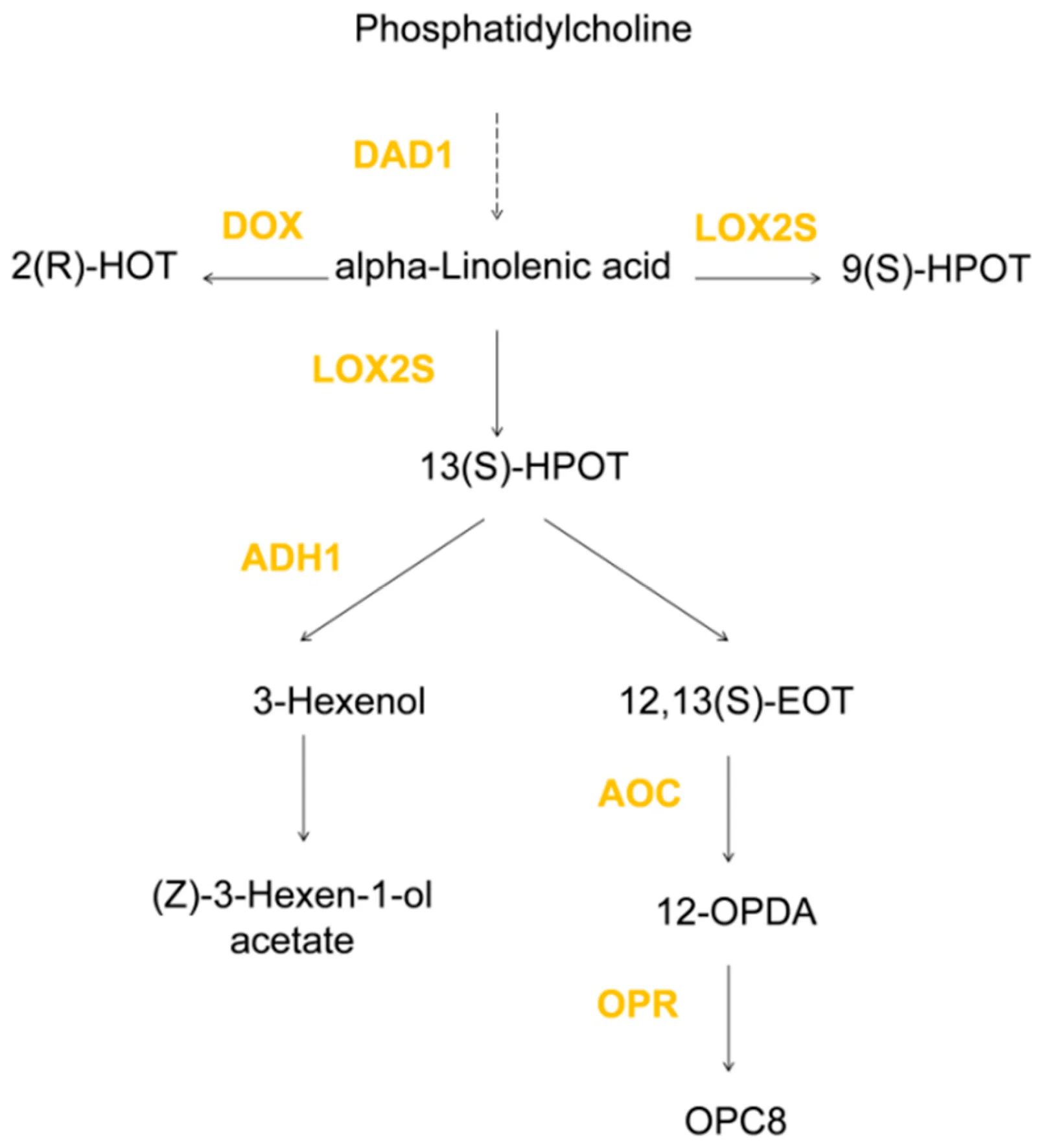
Schematic diagram of the alpha-linolenic acid metabolism pathway and DAPs in alfalfa. The yellow labeled proteins represent DAPs involved in this pathway. Solid arrows indicate single-step enzymatic reactions catalyzed by enzymes labeled in yellow. Dashed arrow represents multi–step reactions with intermediate metabolites omitted from the diagram. DAD1: phospholipase A1; DOX: pathogen–inducible alpha–dioxygenase; LOX2S: lipoxygenase 2S; ADH1: alcohol dehydrogenase 1; AOC: allene oxide cyclase; OPR: putative 12–oxophytodienoate reductase.

Proteomic profiling combined with KEGG enrichment analysis revealed that the DAPs between WT-c and WT-s were predominantly assigned to the alpha-linolenic acid metabolism pathway (Figure 6; Supplementary Table S5). Specifically, a total of six DAPs were assigned to this pathway, and all exhibited marked upregulation in *S. litura* feeding plants relative to the control group (WT-c vs. WT-s). Phospholipase A1 (DAD1, A0A072U2D6) showed a 1.2-fold increase; as a key initiator of JA synthesis, it catalyzes the release of α-linolenic acid from chloroplast membrane lipids, thereby triggering the downstream defense signaling cascade. Pathogen-inducible alpha-dioxygenase (DOX, A0A072U5H0) was upregulated 3.82-fold and cooperates with the lipoxygenase pathway in the generation of green leaf volatiles. Lipoxygenase 2S (LOX2S, G7L7J8), the rate-limiting enzyme in JA biosynthesis responsible for producing 9-HPOT and 13-HPOT substrates, increased by 1.3-fold. Alcohol dehydrogenase 1 (ADH1, A0A072V0U2) was elevated 1.2-fold and participates in the conversion of aldehyde–derived defensive volatiles and the regulation of oxidative stress homeostasis. Allene oxide cyclase (AOC, A0A072TWG0), which catalyzes the formation of the JA precursor 12–OPDA, showed a 1.35-fold increase. Putative 12–oxophytodienoate reductase (OPR, A0A396HPR0) was upregulated 1.42-fold, facilitating the reduction of JA precursors to endogenous JA. Within this pathway, DAD1, LOX2S, AOC, and OPR constitute the core enzymatic cascade for JA biosynthesis, which serves as a primary defense module against herbivorous insects and necrotrophic fungi in plants. The coordinated increases in abundance of these core enzymes in the JA pathway indicate that the feeding of *S. litura* fully activates the JA biosynthesis pathway in alfalfa, which may plausibly contribute to improved defense against *S. litura*.

### 2.7. Analysis of Differentially Abundant Proteins of Alfalfa Related to Photosynthesis During S. litura Feeding

**Figure 7:**
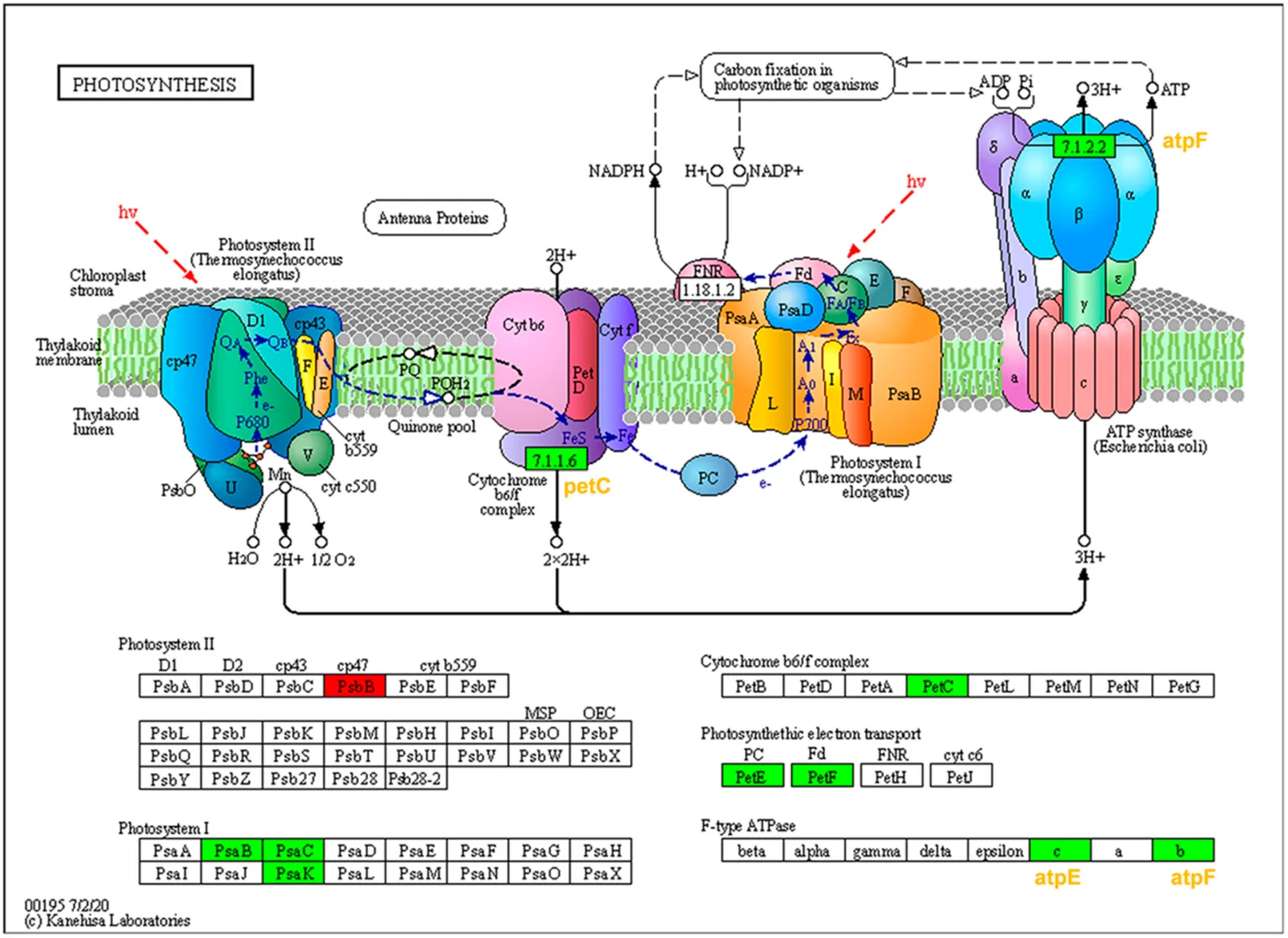
Schematic diagram of the photosynthesis pathway and DAPs in alfalfa. The yellow labeled proteins and the green and red boxes represent the DAPs involved in this pathway. Solid arrows represent direct electron-transfer, proton translocation and one-step biochemical reactions. Dashed arrows indicate metabolite flow connecting to other independent pathways (Calvin–Benson cycle). Red dashed arrows (hν) represent light energy absorption. atpF/ATPF0B: F–type H^+^–transporter ATPase subunit b; atpE/ATPF0C: F–type H^+^–transport ATPase subunit c; petC: iron–sulfur subunit; petE: ferredoxin–NADP^+^ reductase; petF: ferredoxin; psaB: photosystem I P700 chlorophyll a apoprotein A2; psaC: photosystem I iron–sulfur center; psaK: photosystem I subunit X; psbB: photosystem II CP47 chlorophyll apoprotein.

Proteomic analysis combined with KEGG enrichment analysis showed that DAPs between WT-c and WT-s were significantly enriched in the photosynthetic pathway (Figure 7 and Supplementary Table S5). At the same time, after the alfalfa leaves were infested with *S. litura*, the expression levels of various photosynthesis-related proteins decreased, indicating a redistribution of metabolic functions from primary metabolism to defense processes. The abundance of F–type H^+^–transporter ATPase subunit b (ATPF0B) decreased by 0.79 times compared to the control group, which may imply a potential association with chloroplast ATP synthesis and the energy supply for carbon assimilation. The downregulation of F–type H^+^–transport ATPase subunit c (ATPF0C) is more pronounced, with its abundance reduced to 0.55-fold of the control, indicating that the overall ATPase activity is inhibited, thereby affecting the energy supply of photosynthesis. The expression of the iron–sulfur subunit (petC) in the cytochrome b_6_-f complex was also downregulated to 26% of the control, which is likely to hinder the electron transfer between photosystem II (PSII) and photosystem I (PSI). The expression level of ferredoxin–NADP^+^ reductase (petE) dropped to 33% of the control level, which may further limit electron transfer to PSI. Ferredoxin (petF) is a key terminal electron acceptor in the electron transport chain of photosynthesis, and its protein abundance has dropped to 67% of the control group, which may affect the production of downstream NADPH. The large core subunit of photosystem I P700 chlorophyll a apoprotein A2 (psaB) dropped to 0.48-fold, suggesting diminished synthesis of the PSI reaction center proteins and a consequent impairment in electron reception and transfer. The expression of the photosystem I iron–sulfur center (psaC) decreased, reaching 45% of the control value, further affecting the assembly and electron transfer efficiency of PSI complexes. The photosystem I subunit X (psaK) was reduced to 0.79-fold, potentially affecting light capture efficiency and excitation energy transfer within PSI. In contrast to the above components, the photosystem II CP47 chlorophyll apoprotein (psbB) displayed an upregulated trend, with a 1.27-fold increase in the treated group. Overall, the coordinated downregulation of most photosynthesis-associated proteins suggests that *S. litura* feeding may repress the accumulation of photosynthetic machinery in alfalfa, which could potentially result in photodamage or initiate a resource-reallocation strategy within leaf tissues.

## 3. Discussion

The phenylpropanoid pathway acts in a cascade to drive the biosynthesis of lignin, flavonoids, and coumarins, all of which contribute to broad-spectrum resistance and are integral to the plant immune response under biotic stress. PAL, which catalyzes the initial committed step of the pathway, exhibited significantly altered abundance upon *S. litura* feeding, suggesting its potential involvement in phenylpropanoid-mediated herbivore-defense responses. In wheat under aphid attack, PAL activity was significantly upregulated during the feeding period [30]. 4CL contributes to the distribution of metabolic flux among downstream branches. To support this viewpoint, studies on tomatoes demonstrated that silencing *Sl4CLL6* led to increased sensitivity to mites, further emphasizing the functional importance of this enzyme in plant defense [9]. COMT and CCR are the main regulatory factors of lignin biosynthesis. In *Nicotiana attenuata*, both genes were up-regulated due to the feeding of stem borers, and the *PdbCCR*-overexpressing poplar exhibited strong resistance to *Lymantria* dispar larvae [31,32]. In parallel, CHI, FLS, and DFR synergistically guide the accumulation of plant flavonoids, which have been confirmed to possess insect-resistant activities, and these genes were significantly induced in tea upon *Ectropis obliqua* attack and in cassava upon *Phenacoccus manihoti* infestation [33,34]. Moreover, CYP-mediated hydroxylation is a key oxidative modification in the biosynthesis of lignin and flavonoid skeletons. The role of CYP in defense against herbivores has been confirmed via *A. thaliana*-*Pieris rapae* interactions in plants infested by *Cotesia rubecula* and in poplar trees eaten by herbivores [35]. Methylation modifications from O-methyltransferases (OMTs) also helped with plant defense [36]. Functional studies directly demonstrated that overexpression of CCoAOMT enhanced resistance to aphids in sorghum by promoting the accumulation of lignin and phenolic compounds [37]. In addition, in incompatible alfalfa–pathogen interactions, *CHI* transcripts accumulated specifically in infested tissues; nevertheless, the accumulation of phytoalexins (e.g., medicarpin) remained remarkably low, suggesting that transcriptional upregulation does not always translate directly into final product accumulation at the protein or metabolite level and that translational or post-translational regulation may play a critical role [38]. Previous transcriptomic studies reported that the expression of phenylpropanoid biosynthesis-related genes, including *PAL*, *4CL*, *FLS*, and *DFR*, as well as the levels of l-phenylalanine, liquiritigenin, naringenin, and dihydrokaempferol, was significantly upregulated following insect herbivory [29]. In the present study, we found that multiple proteins involved in the phenylpropanoid pathway, namely PAL, CYP73A, COMT, 4CL, OMTs, CCR, CHR, CHI, FLS, and DFR, were consistently upregulated in response to *S. litura* feeding (Figure 4 and Figure 5). These proteomic data effectively complement the limitations of transcriptomic analyses by providing more direct evidence at the protein level, suggesting that the phenylpropanoid biosynthetic pathway plays a pivotal role in alfalfa defense against *S. litura* infestation.

Our proteomic data revealed that DAPs enriched in alpha-linolenic acid metabolism were engaged in JA biosynthesis and oxylipin-mediated signaling, pathways that are centrally positioned in plant defense against wounding and herbivore attacks (Figure 6). These findings are consistent with previous reports identifying DAD1 as a wound-inducible and immunity-related protein. Indeed, transcript induction of *AtDAD1* upon mechanical damage has been documented in *Arabidopsis*, and the orthologous *ZmDAD1* gene in maize was further established as a positive regulator in the defense response to *Bipolaris maydis*, suggesting that DAD1-mediated regulation may represent a conserved component of general stress responses across divergent taxa [39,40]. Plant DOX-catalyzed oxylipin synthesis was previously shown to play an essential role in basal defense against insect herbivory. In *Nicotiana attenuata*, silencing of *Naα-DOX1* substantially impaired resistance to *Manduca sexta*, whereas exogenous 2-HOT supplementation fully rescued the defensive phenotype [41]. LOX2S, a member of the plant lipoxygenase (LOX) family, has been implicated in a broad spectrum of physiological processes, including growth and development, insect resistance, senescence, and wound responses. Functional studies have demonstrated that LOXs confer broad-spectrum resistance against herbivorous insects in both monocotyledonous (e.g., rice and maize) and dicotyledonous (e.g., alfalfa) species, primarily through the regulation of oxylipin biosynthesis [16,29,42]. As an oxidoreductase, alcohol dehydrogenase (ADH) has been suggested to play a regulatory role in the context of fungal pathogenesis and insect host interactions [43]. In rice, the JA biosynthesis and signaling pathway is critically involved in mediating both constitutive and induced defense responses against the striped stem borer (*Chilo suppressalis*) [44]. The rate-limiting nature of 12-oxophytodienoate reductase (OPR) in JA biosynthesis has been substantiated by genetic evidence in multiple species. *ZmOPR2* knockout in maize compromised JA induction and elevated JA catabolites, correlating with enhanced pest susceptibility, and its overexpression in *Arabidopsis* promoted JA accumulation and noctuid defense [45]. In our proteomic dataset, the coordinated upregulation of DAD1, DOX, LOX2S, ADH1, AOC, and OPR strongly suggested that the oxylipin pathway was systemically activated in alfalfa (Figure 6; Supplementary Table S3). However, whether these proteins synergistically drive the biosynthesis of JA or participate in other branches of lipid metabolism remains to be determined through targeted biochemical and genetic validation.

Several photosynthesis-associated proteins exhibited decreased abundance following insect feeding, implying that plants may undergo defensive reprogramming by reallocating carbon and nitrogen resources from growth processes to defensive responses [46]. This phenomenon has been reported in research on the response of various plants such as tobacco and potatoes (*Solanum tuberosum*) to pests. Notably, in potato, feeding by *Leptinotarsa decemlineata* significantly reduced the expression of ATP synthase β subunits and PSI complex subunits (psaE-B), key elements of the photosynthetic electron transport chain [47,48]. In the present study, multiple photosynthesis-related proteins were differentially expressed in alfalfa leaves under *S. litura* feeding (Figure 7). Notably, several subunits of the ATP synthase complex, including the transmembrane proton channel component ATPF0B and the assembly/catalytic subunit ATPF0C, were downregulated in the treated group, suggesting a potential constraint on chloroplastic ATP synthesis efficiency [49]. Consistent with a defense-induced reprogramming of photosynthetic metabolism, the transcript levels of petC (encoding the iron–sulfur protein of the cytochrome b_6_f complex) and petE (plastocyanin) were significantly reduced in response to *S. litura* feeding. Given the role of the b_6_f complex as a redox-state sensor, this downregulation likely attenuated the transmission of redox signals from the electron transport chain [50]. The inhibition of petF further reduces the supply of NADPH, indicating coordinated inhibition of electron flow to the Calvin cycle and other synthetic metabolic pathways, possibly to redirect resources towards defense-related metabolic processes [51]. An obvious exception among these proteins was the accumulation of the PSII core antenna protein PsbB after induction by *S. litura*. Previous studies have shown that damage to cytochrome b_6_f complexes or disruption of electron flow can lead to an imbalance in photosynthetic electron transfer and associated accumulation of reactive oxygen species (ROS), thereby enhancing plant resistance to herbivores [52]. Therefore, we infer that the photo-oxidative microenvironment triggered by PsbB upregulation may establish a local defense barrier at the feeding site, making the tissue less palatable or unsuitable for continuous feeding by *S. litura*. Overall, these proteomic profiles confirm the adaptive regulatory strategy of alfalfa, where metabolic resources are reallocated from primary photosynthesis to defense against herbivores.

Subcellular localization prediction was performed in this study to analyzed the spatial distribution of defense proteins in alfalfa after *S. litura* feeding (Figure 2C). The results revealed that the 586 DAPs were widely distributed across multiple subcellular organelles, including the nucleus, cytoplasm, chloroplasts, mitochondria, and extracellular space, indicating that the defense of alfalfa against herbivores relies on the complex synergistic effects of multiple organelles. This feature is consistent with the protein localization pattern exhibited by perennial grasses when subjected to herbivores [53]. Among these compartments, the cytoplasm (CYT) and extracellular space (EXC) exhibited statistically significant enrichment and functioned as core anti-herbivore-responsive pathways. Previous studies have confirmed that salivary effectors from lepidopteran pests invade plant cells during feeding to disrupt cytoplasmic calcium flux, ROS burst and JA signaling and suppress host immunity. For example, the *Helicoverpa armigera* salivary effector HARP1 targets the cytoplasm and binds JAZ repressors to inhibit JA-mediated defense. In turn, plants specifically upregulate cytoplasmic defense proteins such as calcium-dependent protein kinases, antioxidant enzymes and MAPK signaling proteins to neutralize effector toxicity, which explains the prominent enrichment signature of the CYT compartment in our analysis [54,55]. Moreover, the EXC represented another highly significantly enriched compartment, a phenomenon that has also been reported in herbivore-challenged switchgrass and other crops [53].

## 4. Materials and Methods

### 4.1. Plant Materials

The alfalfa (*Medicago sativa* L. cv. Zhongmu No. 4) independently cultivated by the Chinese Academy of Agricultural Sciences was used in this study. The one-month-old plants used in the experiment were grown in a climate chamber (light: 24 °C/dark: 20 °C; 16 h of light/8 h of darkness; relative humidity: 70%).

### 4.2. Spodoptera litura Rearing and Feeding Assay

The insect used in this experiment is *S. litura*, and the larvae were purchased from Henan Keyun Bio-Pesticides Co., Ltd. (Jiyuan, Henan, China). The rearing method followed previous methods [29]. For the experiment, uniformly sized leaves were collected from the same part of several one-month-old alfalfa plants. The leaves were mixed, and 0.4 g was weighed and placed on a moist filter paper inside an insect-rearing container. The container was maintained at 25 °C, with a light–dark cycle of 16/8 h and a relative humidity of 70%. Experiments were conducted separately for the treatment group and the control group. For the treatment group (WT-s), six third-instar larvae with similar body sizes and weights were starved for 2 h before being placed in each insect-rearing container for continuous treatment for 24 h. For the control group (WT-c), the detached leaves were placed on a moist filter paper in the same environment without adding larvae and were also left for 24 h. Five biological replicates were collected for each treatment, snap-frozen in liquid nitrogen, and stored in a −80 °C freezer. These five harvested samples were pooled, then divided into three equal aliquots for proteomic analysis.

### 4.3. Proteomics Sample Processing and LC-MS/MS Detection

The sample was added to the RIPA lysis buffer, homogenized, and then sonicated on ice (sonication for 3 s with a 3 s interval, for a total of 10 min). It was centrifuged at 12,000× *g* for 10 min at 4 °C, and the supernatant was collected. DTT (final concentration of 10 mmol/L) was added sequentially and reduced at 56 °C for 1 h, followed by the IAM (final concentration of 55 mmol/L) reaction in the dark for 40 min. Six volumes of precooled acetone were added, and the mixture was precipitated overnight at −20 °C. It was centrifuged at 10,000× *g* for 10 min at 4 °C; the acetone was discarded, and the precipitate was dried for 2–3 min. The precipitate was redissolved with 100 μL of 50 mmol/L NH_4_HCO_3_; then 1 μg of trypsin was added, and enzymatic digestion was performed overnight at 37 °C. The digested peptides were desalted, evaporated to dryness under vacuum, and redissolved with 0.1% formic acid, and the supernatant was collected by centrifugation for mass spectrometry analysis.

LC-MS/MS analysis was performed using a Vanquish liquid chromatograph (Thermo Fisher Scientific, Waltham, MA, USA) and an Orbitrap Astral mass spectrometer (Thermo Fisher Scientific, Waltham, MA, USA). LC-MS/MS measurements were performed in DIA mode on a 100 μm i.d. × 180 mm capillary column packed with 3 μm Reprosil-Pur 120 C18-AQ particles (Dr. Maisch GmbH, Ammerbuch, Germany) using mobile phases A (0.1% formic acid-water) and B (0.1% formic acid in 80% acetonitrile) at 600 nL/min. MasterScan spectra were acquired at 240,000 resolving power across 380–980 *m*/*z*, while DIA fragmentation adopted an isolation width of 2 *m*/*z*, 299 sequential scan events and a stepped NCE of 25, and uniform instrument settings were used for all samples to guarantee experimental repeatability. The raw mass spectrometry data were searched against a corresponding species protein database using Spectronaut 14.2 software (Biognosys AG, Schlieren, Switzerland). Quantification was performed using a DIA-based label-free relative quantification technique and analyzed using DIA-NN software (v 1.9) [56]. Based on the total peak area of all proteins in each sample, the peak area of each protein was normalized by dividing it by the total peak area of that sample.

### 4.4. Differentially Abundant Protein Analysis and Functional Annotation

The screening of DAPs was performed using a combination threshold method. Using fold change (FC) and *t*-test *p*-values as screening indicators, the specific criteria are as follows: when FC ≥ 1.2 and *p* ≤ 0.05, it is determined to be an upregulated expressed protein; when FC ≤ 1/1.2 and *p* ≤ 0.05, it is determined to be a downregulated expressed protein. This moderate fold-change threshold was applied in combination with statistical filtering, and biological interpretation mainly relies on pathway-level enrichment rather than individual protein fluctuations. We use ggplot2 package (v3.3.6) [57] to draw a volcano plot and display the overall distribution of DAPs. DAP hierarchical clustering was analyzed using heatmaps to evaluate their expression patterns in each sample, and the analysis and visualization were carried out through TBtools-II software (v2.458) [58].

For the identified DAPs, the GO (Gene Ontology, http://geneontology.org/, accessed on 25 May 2026), KEGG (Kyoto Encyclopedia of Genes and Genomes enrichment analysis, http://www.genome.jp/kegg/, accessed on 25 May 2026) and COG (Clusters of Orthologous Groups of proteins) [59] databases were used for functional annotations and to understand the functional characteristics of DAPs.

### 4.5. Structure and Localization Prediction of Differentially Abundant Proteins

The Pfam (Protein families database) [60] database was used to predict the structural domains of proteins. Subcellular localization enrichment of DAPs was predicted with the machine-learning-based tool ng_Loc v1.0, using the plant protein benchmark dataset from Swiss-Prot as the background dataset. The enrichment ratio was defined as the ratio between the percentage of DAPs localized to one subcellular compartment and the percentage of all quantified proteins assigned to the same compartment. A total of 586 DAPs were subjected to enrichment analysis and significantly enriched subcellular localization categories were screened using *p*-values.

### 4.6. Statistical Analysis

Graph plotting was completed using GraphPad Prism 7 software (GraphPad Software, San Diego, CA, USA). Statistical analysis was performed using one-way analysis of variance (ANOVA) in SPSS 19.0 software, supplemented by Duncan’s test for multiple comparisons. The significance level was set at 0.05, and different lowercase letters in the figure indicate significant differences between treatment groups (*p* < 0.05).

## 5. Conclusions

Proteomic analyses reveal that alfalfa exhibits alterations in protein abundance across multiple pathways upon exposure to *S. litura* feeding stress. Herbivory induces increased abundance of key enzymes associated with phenylpropanoid and flavonoid biosynthesis, which may promote the biosynthesis of lignin- and flavonoid-related defensive compounds, and also elevates the abundance of core proteins involved in JA signaling. These two sets of protein changes may contribute to alfalfa’s response to biotic stress. In addition, *S. litura* feeding reduces the abundance of photosynthesis-associated proteins; such protein-level alterations may potentially compromise the photosynthetic machinery and drive putative resource reallocation from primary photosynthetic metabolism toward defensive secondary metabolism. Collectively, our proteomic data indicate that insect feeding triggers metabolic reprogramming in alfalfa via altered abundance of proteins associated with defensive secondary and primary photosynthetic metabolism, and the candidate proteins and defense-related pathways identified here provide potential molecular resources for future improvement of alfalfa insect resistance and may inform plant-protection strategies for alfalfa agro-ecosystems.

## Data Availability

The mass spectrometry proteomics data from this study have been uploaded and stored in the iProX repository with the dataset identifier IPX0019027000. The ProteomeXchange registration number is PXD082672. The dataset has not been released and will be publicly available upon reasonable request.

## Supplementary Materials

The following supporting information can be downloaded at https://www.mdpi.com/article/10.3390/plants15193064/s1, Supplementary Figure S1: Functional classification of DAPs; Supplementary Figure S2: KEGG functional annotation of DAPs; Supplementary Table S1: Protein information of all samples; Supplementary Table S2: Protein information of all DAPs; Supplementary Table S3: Top 20 significantly enriched GO terms in GO annotation analysis; Supplementary Table S4: Top 20 significantly enriched KEGG terms in KEGG annotation analysis; Supplementary Table S5: Important DAP information and protein abundance under *S. litura* feeding.

## References

[1] Desta A.G. (2026). The role of alfalfa (*Medicago sativa* L.) in soil health and greenhouse gas mitigation in integrated crop-livestock systems: A review. Arch. Agron. Soil Sci..

[2] Capinera J.L., Capinera J.L. (2008). Alfalfa (Lucerne) pests and their management. Encyclopedia of Entomology.

[3] Cheng T.C., Wu J.Q., Wu Y.Q., Chilukuri R.V., Huang L.H., Yamamoto K., Feng L., Li W.S., Chen Z.W., Guo H.Z. (2017). Genomic adaptation to polyphagy and insecticides in a major East Asian noctuid pest. Nat. Ecol. Evol..

[4] Mallikarjuna N., Kranthi K.R., Jadhav D.R., Kranthi S., Chandra S. (2004). Influence of foliar chemical compounds on the development of *Spodoptera litura* (Fab.) in interspecific derivatives of groundnut. J. Appl. Entomol..

[5] Yan J., Qiu R., Wang K., Liu Y., Zhang W. (2023). Enhancing alfalfa resistance to *Spodoptera* herbivory by sequestering microRNA396 expression. Plant Cell Rep..

[6] Vasantha-Srinivasan P., Noh M.Y., Park K.B., Kim T.Y., Jung W.J., Senthil-Nathan S., Han Y.S. (2025). Plant immunity to insect herbivores: Mechanisms, interactions, and innovations for sustainable pest management. Front. Plant Sci..

[7] Zhu L.H., Yang H.C., Li P.X., Dong L.N., Zhao S., Lv H.H., Crickmore N., Zhou X.G., Zhang Y.J., Guo Z.J. (2026). Plant strategies against herbivorous insects. J. Integr. Plant Biol..

[8] Zhang Z.Q., Chen Q., Sa R., Dai R.L., Shuang S., Jiang X.H., Liu H.J., Tan Y., Tang F., Temuer B. (2022). Morphological and biochemical factors associated with constitutive defense to thrips in alfalfa. Agronomy.

[9] Wang M.L., Wang Y.D., Li X.Z., Zhang Y., Chen X.L., Liu J.Y., Qiua Y.W., Wang A.X. (2024). Integration of metabolomics and transcriptomics reveals the regulation mechanism of the phenylpropanoid biosynthesis pathway in insect resistance traits in *Solanum habrochaites*. Hortic. Res..

[10] Chen M.S. (2008). Inducible direct plant defense against insect herbivores: A review. Insect Sci..

[11] Liu X.B., Zhang Y.M., Francis F., Cheng Y.M., Liu Y.X., Chen J.L., Zhang Y. (2026). Induced Bowman-Birk trypsin inhibitor *TaBBTI-1* in wheat during poor-host interactions with aphids confers broad-spectrum resistance to insect herbivores. Int. J. Biol. Macromol..

[12] Roy R., Kerns D., Jurat-Fuentes J.L. (2025). Resistance to Vip3Aa: A growing threat with unclear mechanisms and management implications. Insects.

[13] Mo H.J., Chang H.M., Zhao G., Hu G.J., Luo X.M., Jia X., Xu Z.L., Ren G.M., Feng L., Wendel J.F. (2024). iJAZ-based approach to engineer lepidopteran pest resistance in multiple crop species. Nat. Plants.

[14] Keerthana M., Reddy M.S.S., Naik Y.D., Pal A., Kale A.N., Singh S.K., Nandni S., Premalatha K., Jayakodi M., Mendu V. (2026). Fusion protein based strategies for developing insect-resistant crops. Funct. Integr. Genom..

[15] Zhang L., Zhang F., Melotto M., Yao J., He S.Y. (2017). Jasmonate signaling and manipulation by pathogens and insects. J. Exp. Bot..

[16] Liao Z.H., Wang L., Li C.Z., Cao M.J., Wang J.N., Yao Z.L., Zhou S.Y., Zhou G.X., Zhang D.Y., Lou Y.G. (2022). The lipoxygenase gene *OsRCI-1* is involved in the biosynthesis of herbivore-induced JAs and regulates plant defense and growth in rice. Plant Cell Environ..

[17] Ye M., Luo S.M., Xie J.F., Li Y.F., Xu T., Liu Y., Song Y.Y., Zhu-Salzman K., Zeng R.S. (2012). Silencing COI1 in rice increases susceptibility to chewing insects and impairs inducible defense. PLoS ONE.

[18] Yang J., Duan G.H., Li C.Q., Liu L., Han G.Y., Zhang Y.L., Wang C.M. (2019). The crosstalks between jasmonic acid and other plant hormone signaling highlight the involvement of jasmonic acid as a core component in plant response to biotic and abiotic stresses. Front. Plant Sci..

[19] Lü S.Y., Zhu Z.H., Yu X.Y., Dong C.Y., Cao J.M., Zhang Z.Y., Liu H.F., Hu Q., Hu J., Hu Q. (2025). Phenylpropanoid metabolites mediate antiviral defense and vector resistance in rice infected with RRSV, RGSV, and SRBSDV. Plant Cell Environ..

[20] Yao S., Zhang T., Guo C.M., Sha S., Mu K.L., Zhang Z.W., Luo Q.M., Pang Y.X. (2026). Exogenous methyl jasmonate enhances chemical defense in blumea balsamifera against *Spodoptera litura* by boosting phenylpropanoid and flavonoid metabolism. Int. J. Mol. Sci..

[21] Ninkuu V., Aluko O.O., Yan J.P., Zeng H.M., Liu G.D., Zhao J., Li H.H., Chen S.B., Dakora F.D. (2025). Phenylpropanoids metabolism: Recent insight into stress tolerance and plant development cues. Front. Plant Sci..

[22] Yang Y.N., Liu M., Huang Z.H. (2024). Genomic and expression analysis of cassava (*Manihot Esculenta Crantz*) chalcone synthase genes in defense against *Tetranychus cinnabarinus* infestation. Genes.

[23] Liu H., Wang X.Z., Ma Y., Gao W.S., Wang X.J., Ma C.H. (2025). Dual defense strategies in alfalfa: A multi-omics investigation into differential activation of defense pathways against adult and larval weevil feeding. Ind. Crops Prod..

[24] Batyrshina Z.S., Yaakov B., Shavit R., Singh A., Tzin V. (2020). Comparative transcriptomic and metabolic analysis of wild and domesticated wheat genotypes reveals differences in chemical and physical defense responses against aphids. BMC Plant Biol..

[25] Zhang Z.Q., Chen Q., Tan Y., Shuang S., Dai R., Jiang X.H., Temuer B. (2021). Combined transcriptome and metabolome analysis of alfalfa response to thrips infection. Genes.

[26] Rustagi A., Chugh S., Sharma S., Kumari P., Kumar D., Singh I.K., Singh A. (2021). Plant-Insect Interaction: A proteomic approach in defence mechanism. Plant-Pest Interactions: From Molecular Mechanisms to Chemical Ecology.

[27] Revanayya G., Singh I., Dhanyakumar O., Jaba J., Bhatia D., Bindra S., Singh R., Kudapa H., Yogendra K. (2026). Comparative proteomics provides insights into activation of jasmonic acid-mediated resistance mechanism in chickpea—*Helicoverpa armigera* interaction. Plant Cell Rep..

[28] Li S., Tan X., Yang L., Deng X., Li M., Qin L., Qi L., Li J., Luo G., Yuan M. (2025). Global profiling of lysine-malonylated proteins in rice elucidates the immune-regulatory mechanisms of malonylation under herbivore or viral stresses. J. Integr. Plant Biol..

[29] Xu L., Li M.N., Xu Y.C., Xu Y.R., Yu H.G., He F., Kang J.M., Yang H.S., Yuan F., Zhang Y.R. (2026). Overexpression of *MtLOX24* enhances alfalfa resistance to *Spodoptera litura* via phenylpropanoids and jasmonic acid pathways. Plant Physiol. Biochem..

[30] Xu Y., Guo H., Geng G.D., Zhang Q.Q., Zhang S.Q. (2021). Changes in defense-related enzymes and phenolics in resistant and susceptible common wheat cultivars under aphid stress. Acta Physiol. Plant.

[31] Joo Y., Kim H., Kang M., Lee G., Choung S., Kaur H., Oh S., Choi J.W., Ralph J., Baldwin I.T. (2021). Pith-specific lignification in *Nicotiana attenuata* as a defense against a stem-boring herbivore. New Phytol..

[32] Li Y., Zhang R.Q., Sun L.L., Cao C.W. (2025). Resistance of *Populus davidiana* × *P. bolleana* overexpressing *cinnamoyl-CoA* reductase gene to *Lymantria dispar* larvae. Transgenic Res..

[33] Wang Y.N., Tang L., Hou Y., Wang P., Yang H., Wei C.L. (2016). Differential transcriptome analysis of leaves of tea plant (*Camellia sinensis*) provides comprehensive insights into the defense responses to *Ectropis oblique* attack using RNA-Seq. Funct. Integr. Genom..

[34] Geng Y., Chen Q., Liang X., Liu Y., Wu C.L., Chen Y.H. (2023). Flavonoid synthesis pathway-mediate defense mechanism of cassava against *Phenacoccus manihoti*. Chin. J. Trop. Crop.

[35] Singh A., Panwar R., Mittal P., Hassan M.I., Singh I.K. (2021). Plant cytochrome P450s: Role in stress tolerance and potential applications for human welfare. Int. J. Biol. Macromol..

[36] Kim B.G., Yi Y. (2009). Flavonoid methylation of poplar O-Methyltransferase encoded in *Escherichia coli*. J. Korean Soc. Appl. Biol. Chem..

[37] Ikuze E., Grover S., Puri H., Kundu P., Sattler S., Louis J. (2025). Overexpression of the sorghum *CCoAOMT* gene confers enhanced resistance to sugarcane aphids. Physiol. Plant.

[38] Sallaud C., Zuanazzi J., El-Turk J., Leymarie J., Breda C., Buffard D., Kozak I., Ratet P., Husson P., Kondorosi A. (1997). Gene expression is not systematically linked to phytoalexin production during alfalfa leaf interaction with pathogenic bacteria. Mol. Plant Microbe Interact..

[39] Ruduś I., Terai H., Shimizu T., Kojima H., Hattori K., Nishimori Y., Tsukagoshi H., Kamiya Y., Seo M., Nakamura K. (2014). Wound-induced expression of *DEFECTIVE IN ANTHER DEHISCENCE1* and DAD1-like lipase genes is mediated by both *CORONATINE INSENSITIVE1*-dependent and independent pathways in *Arabidopsis thaliana*. Plant Cell Rep..

[40] Lv R.Y., Wu H.L., Yu H.Y., Ning N., Liu C.T., Ding Y., Li S., Liu W.D., Li Z.Q. (2026). Positive regulation of maize resistance to *Bipolaris maydis* by the cell death suppressor *ZmDAD1*. Plant Physiol. Biochem..

[41] Gaquerel E., Steppuhn A., Baldwin I.T. (2012). *Nicotiana attenuata* α-DIOXYGENASE1 through its production of 2-hydroxylinolenic acid is required for intact plant defense expression against attack from *Manduca sexta* larvae. New Phytol..

[42] Yuan P.G., Borrego E., Park Y., Gorman Z., Huang P.C., Tolley J., Christensen S.A., Blanford J., Kilaru A., Meeley R. (2023). 9,10-KODA, an α-ketol produced by the tonoplast-localized 9-lipoxygenase *ZmLOX5*, plays a signaling role in maize defense against insect herbivory. Mol. Plant.

[43] Gutiérrez-Corona J.F., González-Hernández G.A., Padilla-Guerrero I.E., Olmedo-Monfil V., Martínez-Rocha A.L., Patiño-Medina J.A., Meza-Carmen V., Torres-Guzmán J.C. (2023). Fungal alcohol dehydrogenases: Physiological function, molecular properties, regulation of their production, and biotechnological potential. Cells.

[44] Danso Ofori A., Su W., Zheng T.D., Datsomor O., Titriku K.J., Xiang X., Kandhro A.J., Ahmed M.I., Mawuli E.W., Awuah R.T. (2025). Jasmonic acid (JA) signaling pathway in rice defense against *Chilo suppressalis* infestation. Rice.

[45] Huang P.C., Grunseich J.M., Berg-Falloure K.M., Tolley J.P., Koiwa H., Bernal J.S., Kolomiets M.V. (2023). Maize OPR2 and LOX10 mediate defense against fall armyworm and western corn rootworm by tissue-specific regulation of jasmonic acid and ketol metabolism. Genes.

[46] Zhang B.C., Zhou L.Y., Zhou X.H., Bai Y.F., Zhan M.K., Chen J., Xu C.Y. (2022). Differential responses of leaf photosynthesis to insect and pathogen outbreaks: A global synthesis. Sci. Total Environ..

[47] Hermsmeier D., Schittko U., Baldwin I.T. (2001). Molecular interactions between the specialist Herbivore *Manduca sexta* (Lepidoptera, Sphingidae) and its natural host *Nicotiana attenuata*. I. Large-scale changes in the accumulation of growth- and defense-related plant mRNAs. Plant Physiol..

[48] Duceppe M.O., Cloutier C., Michaud D. (2012). Wounding, insect chewing and phloem sap feeding differentially alter the leaf proteome of potato, *Solanum tuberosum* L.. Proteome Sci..

[49] Yue X.H., Ke X.S., Shi Y.F., Li Y.S., Zhang C.H., Wang Y.T., Hou X. (2023). Chloroplast inner envelope protein FtsH11 is involved in the adjustment of assembly of chloroplast ATP synthase under heat stress. Plant Cell Environ..

[50] Malone L.A., Proctor M.S., Hitchcock A., Hunter C.N., Johnson M.P. (2021). Cytochrome *b_6_f*–Orchestrator of photosynthetic electron transfer. BBA–Bioenerg..

[51] Sawyer A., Winkler M. (2017). Evolution of Chlamydomonas reinhardtii ferredoxins and their interactions with [FeFe]-hydrogenases. Photosynth. Res..

[52] Lu J.Z., Yin Z.P., Lu T., Yang X.L., Wang F., Qi M.F., Li T.L., Liu Y.F. (2020). Cyclic electron flow modulate the linear electron flow and reactive oxygen species in tomato leaves under high temperature. Plant Sci..

[53] Zogli P., Alvarez S., Naldrett M.J., Palmer N.A., Koch K.G., Pingault L., Bradshaw J.D., Twigg P., Heng-Moss T.M., Louis J. (2020). Greenbug (*Schizaphis graminum*) herbivory significantly impacts protein and phosphorylation abundance in switchgrass (*Panicum virgatum*). Sci. Rep..

[54] Chen C.Y., Liu Y.Q., Song W.M., Chen D.Y., Chen F.Y., Chen X.Y., Chen Z.W., Ge S.X., Wang C.Z., Zhan S. (2019). An effector from cotton bollworm oral secretion impairs host plant defense signaling. Proc. Natl. Acad. Sci. USA.

[55] Parmagnani A.S., Maffei M.E. (2022). Calcium signaling in plant-insect interactions. Plants.

[56] Demichev V., Messner C.B., Vernardis S.I., Lilley K.S., Ralser M. (2020). DIA-NN: Neural networks and interference correction enable deep proteome coverage in high throughput. Nat. Methods.

[57] Wickham H. (2011). ggplot2. WIREs Comp. Stat..

[58] Chen C.J., Chen H., Zhang Y., Thoma H.R., Frank M.H., He Y.H., Xia R. (2020). TBtools: An integrative toolkit developed for interactive analyses of big biological data. Mol. Plant..

[59] Tatusov R.L., Fedorova N.D., Jackson J.D., Jacobs A.R., Kiryutin B., Koonin E.V., Krylov D.M., Mazumder R., Mekhedov S.L., Nikolskaya A.N. (2003). The COG database: An updated version includes eukaryotes. BMC Bioinform..

[60] Bateman A., Coin L., Durbin R., Finn R.D., Hollich V., Griffiths-Jones S., Khanna A., Marshall M., Simon M., Erik L.L. (2004). The Pfam protein families database. Nucleic Acids Res..

