# Supplementary material for: Proteomic Profiling of Medicago sativa Leaves upon Spodoptera litura (Lepidoptera: Noctuidae) Invasion: Activation of Defense Pathways and Photosynthetic Resource Reallocation
Source: Plants (Basel). 2026 Oct 7;15(19):3064. doi: 10.3390/plants15193064 (PMC13666713; doi:10.3390/plants15193064)
Supplement: Supplementary file 1 [file plants-15-03064-s001.zip › Supplementary Figure.pdf]

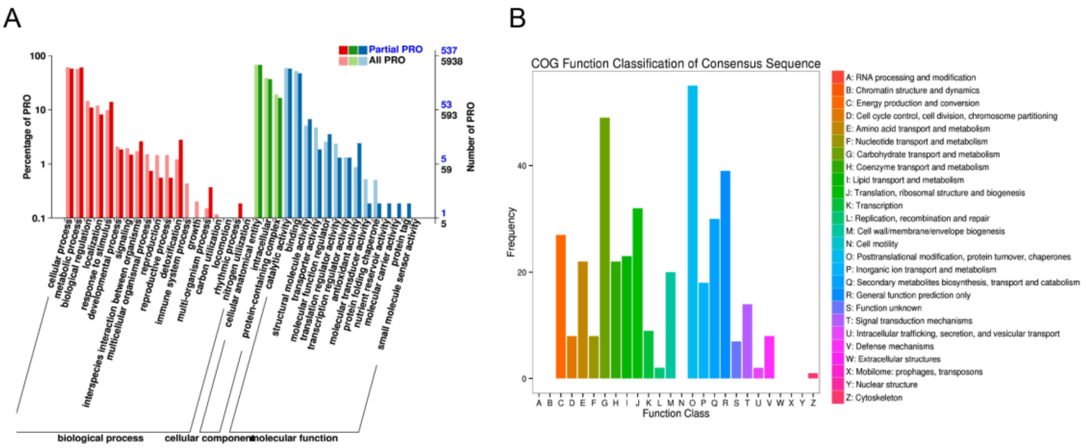

Figure S1. Functional classification of DAPs. (**A**) GO classification of DAPs. The x-axis represents specific GO terms, the left y-axis indicates the percentage of DAPs annotated to each term relative to the total number of DAPs, and the right y-axis denotes the number of DAPs annotated to each term. (**B**) Bar chart of COG (Clusters of Orthologous Groups) classification of DAPs. The x-axis stands for COG categories, and the y-axis represents the number of DAPs annotated to each corresponding category.

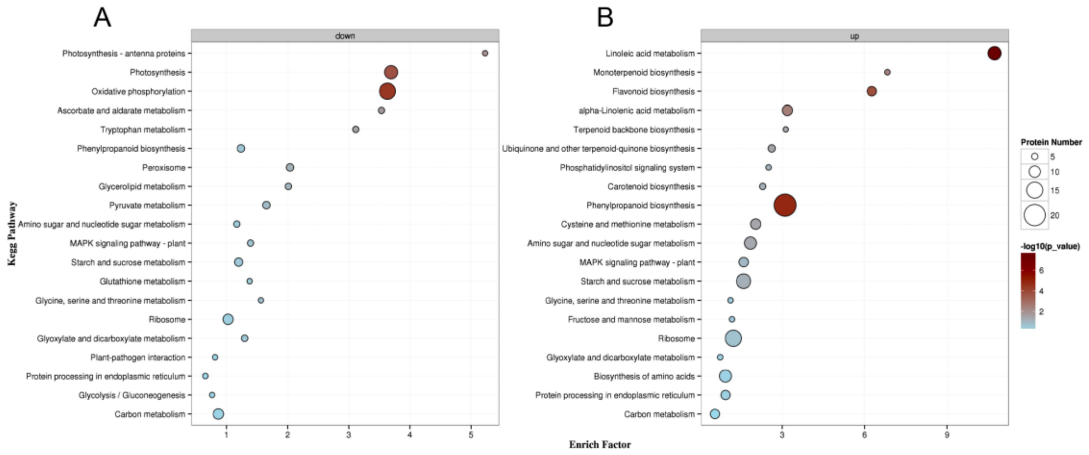

Figure S2. KEGG functional annotation of DAPs. (**A**) Bubble chart of KEGG enrichment for downregulated DAPs. (**B**) Bubble chart of KEGG enrichment for upregulated DAPs. Each dot denotes a KEGG pathway; the y-axis and x-axis represent pathway names and enrichment factors, respectively.
